# Comparison of Self-Reported Alcohol Consumption to Phosphatidylethanol Measurement among HIV-Infected Patients Initiating Antiretroviral Treatment in Southwestern Uganda

**DOI:** 10.1371/journal.pone.0113152

**Published:** 2014-12-01

**Authors:** Francis Bajunirwe, Jessica E. Haberer, Yap Boum, Peter Hunt, Rain Mocello, Jeffrey N. Martin, David R. Bangsberg, Judith A. Hahn

**Affiliations:** 1 Mbarara University of Science and Technology, Faculty of Medicine, Department of Community Health, P.O. Box 1410, Mbarara, Uganda; 2 Harvard Medical School, Boston, Massachusetts, United States of America; 3 Epicentre Uganda Research Center, Mbarara, Uganda; 4 Department of Epidemiology and Biostatistics, University of California San Francisco, San Francisco, California, United States of America; 5 Department of Medicine, University of California San Francisco, San Francisco, California, United States of America; University of Missouri-Kansas City, United States of America

## Abstract

**Background:**

Alcohol consumption among HIV-infected patients may accelerate HIV disease progression or reduce antiretroviral therapy adherence. Self-reported alcohol use is frequently under-reported due to social desirability and recall bias. The aim of this study was to compare self-reported alcohol consumption to phosphatidylethanol (PEth), a biomarker of alcohol consumption, and to estimate the correlation between multiple measures of self-reported alcohol consumption with PEth.

**Methods:**

The Uganda AIDS Rural Treatment Outcomes (UARTO) cohort is located in southwestern Uganda and follows patients on ART to measure treatment outcomes. Patients complete standardized questionnaires quarterly including questions on demographics, health status and alcohol consumption. Baseline dried blood spots (DBS) were collected and retrieved to measure PEth.

**Results:**

One hundred fifty samples were tested, and 56 (37.3%) were PEth positive (≥8 ng/mL). Of those, 51.7% did not report alcohol use in the past month. Men were more likely to under-report compared to women, OR 2.9, 95% CI = 1.26, 6.65) and those in the higher economic asset categories were less likely to under-report compared to those in the lowest category (OR = 0.41 95% CI: 0.17, 0.94). Among self-reported drinkers (n = 31), PEth was highly correlated with the total number of drinking days in the last 30 (Spearman R = 0.73, p<0.001).

**Conclusions:**

Approximately half of HIV infected patients initiating ART and consuming alcohol under-report their use of alcohol. Given the high prevalence, clinicians should assess all patients for alcohol use with more attention to males and those in lower economic asset categories who deny alcohol use. Among those reporting current drinking, self-reported drinking days is a useful quantitative measure.

## Introduction

Uganda faces a dual burden of HIV and unhealthy alcohol use. The country is ranked among the top per capita consumers of alcohol in the world [1] and also has a high prevalence of HIV of 7.3% among the adults [2]. This dual burden of heavy alcohol use and HIV may present challenges because alcohol use among HIV patients has been associated with increased progression of HIV disease [3], potentially due to the effect of alcohol on the immune system [4]. Alcohol is known to interact with certain antiretroviral treatments hence reducing treatment efficacy [5], and alcohol consumption is associated with poor adherence to antiretroviral medication [6]–[8] and with excess mortality [9]–[11]. Effective treatments in the form of behavioral or pharmacological therapy [12] exist to reduce the negative effects of alcohol among those with alcohol use disorders; while screening and brief interventions may reduce alcohol consumption among problem drinkers who are not alcohol dependent [13].

To identify patients who consume alcohol, counselors and clinicians often rely on self-reported measures of alcohol consumption which may be biased toward under-report by social desirability to report what the counselor or clinician wants to hear, or out of fear of being denied ART [14] or simply recall bias. The frequency of under-reporting has been explored in very few studies, however in a nested case control from our cohort, the frequency of self-reported prior 3-month alcohol use increased from 20% to 41% when alcohol biomarker and breathalyzer testing was included as part of the study protocol and discussed during the informed consent process [15]. There are also challenges in quantifying alcohol consumption in resource limited settings such as Uganda, where drinks are often served in non-standard containers such as gourds or consumed communally. In order to accurately measure alcohol consumption, biomarkers of alcohol such as phosphatidylethanol (PEth), a phospholipid which is formed by the action of a phospholipase enzyme only in the presence of alcohol, may be useful. PEth has been tested in Uganda and shown to be highly sensitive (88%) and specific (89%) for any alcohol consumption in the prior 21 days [16]. Thus this biomarker provides a tool to estimate under report.

The aim of this study was to determine the frequency of alcohol under-reporting among patients receiving HIV treatment, the characteristics of the patients who underreport, and examine the correlation of quantitative PEth results with various self-reported measures of alcohol consumption in rural Uganda.

## Methods

### Setting and study procedures

Participants in this study were part of the Uganda AIDS Rural Treatment Outcomes (UARTO) cohort study located in Mbarara, which is in southwestern Uganda. The UARTO cohort began in July 2005 and recruited treatment-naïve patients initiating ART at Mbarara Hospital's HIV clinic. A study representative approached individuals collecting new antiretroviral prescriptions from the pharmacy, and determined their eligibility and interest in participation. Those who were at least 18 years of age and lived within 50 kilometers of the clinic were eligible for the study. The cohort is fully recruited now but follow-up is still ongoing.

### Ethics statement

All study procedures were approved by the Institutional review Boards of the Mbarara University of Science and Technology, Partners HealthCare, the University of California, San Francisco, and the Uganda National Council of Science and Technology. All participants enrolled in the study provided written informed consent. As part of the consent process, participants agreed to have their blood samples stored, and were told that the blood may be tested to see if they had consumed alcohol. Upon recruitment, UARTO participants underwent a baseline interview and phlebotomy. Participants completed standardized interviewer-administered surveys detailing demographics, household socioeconomic profile, alcohol use, mental and physical health status and depression scores among other variables, as well as CD4 cell count and HIV RNA quantification, on a quarterly basis.

### Measurement of alcohol consumption

We asked study participants when they last consumed alcohol, and how many days in the prior 30 they consumed alcohol. We additionally asked the typical number of drinks consumed per drinking day for those drinking alcohol in forms other than gourds or non-standard drink containers. The number of drinks per day was used to calculate the total number of drinks in the last 30 days. We asked the total amount of money (in Ugandan shillings) that the participants spent on their own alcohol in the prior 30 days. Lastly, we asked the number of days the participants drank until they reached the following stages of intoxication (in descending order of severity): drinking until feeling stuporous or becoming unconscious; drinking until it was difficult to speak or see clearly or walk; drinking until it was difficult to think clearly; and drinking until feeling uninhibited or a false sense of confidence. We added the number of days the participants reported these to create a variable representing the number of days of drinking until intoxication.

### Covariates

Depression was assessed using the 15-items of the Hopkins Symptoms checklist depression scale (HSCL) that screen for depression [17]. The tool has been used widely in the region and has been found to have high validity and reliability [18]. We used a cut-off of average value of greater than or equal to 1.75 to indicate a positive screen for clinical depression. Literacy levels were assessed using simple reading cards in the local language and English. Socio-economic status was assessed using education, electricity in the home, asset category and land ownership. Economic asset categories to indicate household wealth were constructed using Principal components analysis [19]. Low represents the bottom two quintiles and the rest were classified in a single category representing medium to high assets.

#### Dried blood spot collection and phosphatidylethanol testing

Phospatidylethanol (PEth) is a metabolite of alcohol and is a highly sensitive and specific biomarker for alcohol consumption and has been shown to have the highest sensitivity for detecting alcohol intake over the last 21 days compared to the other biomarkers such as carbohydrate deficient transferin (CDT), mean corpuscular volume (MCV) and gamma-glutamyl transferase [20]. In a previous study of persons with HIV attending the Mbarara Hospital HIV Clinic different from those enrolled in the current study, we found that PEth was 88% sensitive and 89% specific for any alcohol consumption in the prior 21 days. [16] In that study any alcohol consumption was defined as either detectable alcohol use on any daily breathalyzer test conducted at home or a pre-arranged drinking establishment, or any and alcohol use self-reported on a daily survey.

In addition, the study found that PEth was highly correlated with several quantitative measures of alcohol consumption, such as number of days drinking (Spearman r = 0.74); others have shown similar results in HIV uninfected populations. [21], [22]

We collected dried blood spots (DBS) at all study visits beginning July 2011, and retrieved those for the baseline visits for the first 150 patients in August 2013, therefore samples were stored for a maximum of two years. PEth testing was conducted at the United States Drug Testing Laboratory, Des Plaines, IL, using a previously published method. [23] PEth was detected in standard dried blood spot punches (3.1 mm) using an Agilent 6460 liquid chromatography-tandem mass spectrometry (LC-MS/MS) system following extraction into methanol. The most prevalent PEth isomer, palmitoyl (PEth 16∶0)/oleoyl (PEth 18∶1), was detected. Positive tests were confirmed with a repeat test, and the average of the two results was used. The limit of detection was 2 ng/mL, the limit of quantitation was 8 ng/mL, and the assay was linear up to 800 ng/mL.

### Statistical analysis

We calculated the proportions for categorical variables and means or medians for continuous variables. We determined the sensitivity of self-reported alcohol use compared to PEth. Participants were considered to have under-reported alcohol consumption if they tested positive for PEth (≥8 ng/mL, the current limit of quantification) but report no alcohol consumption in the last 30 days. We use logistic regression to determine the factors associated with under-reporting. We conducted 3 sets of logistic regression in which the outcome variable was a PEth positive result. These regressions were conducted (1) among all participants, (2) among those who reported prior 30 day drinking and/or those who were PEth positive, collectively called drinkers, and (3), among those who reported no drinking in the prior 30 days. We used the Wilcoxon rank sum test (Mann-Whitney) to compare the median PEth values of those who did to those who did not report any 30 day drinking, overall and by sex, among those who tested positive for PEth. Lastly, we calculated Spearman's rank correlations between the quantitative PEth values and several self-reported measures of alcohol consumption among those who reported any 30 day drinking.

## Results

### Baseline characteristics

Of the 150 participants, 65% were female, 43% were aged 31 to 45 years and 53% were of Protestant religion (Table 1). Almost one quarter (23%) of the respondents could not read a sentence and 26% screened positive for depression.

**Table 1:** Baseline characteristics (n = 150).

Characteristic	N (%)
Gender	
Male	52 (34.6)
Female	98 (65.4)
Age	
18–30	63 (42.0)
31–45	65 (43.3)
Over 45	22 (14.7)
Religion	
Protestant	79 (52.7)
Catholic	45 (30.0)
Moslem	17 (11.3)
Pentecostal	9 (6.0)
Education	
None	28 (18.6)
Primary	78 (52.0)
Secondary and above	44 (29.3)
Own land	
No	63 (42.0)
Yes	87 (58.0)
Literacy	
Cannot read at all	34 (23.3)
Able to read parts of a sentence	26 (17.8)
Able to read whole sentence	86 (58.9)
Electricity in the home	
Yes	38 (25.4)
No	112 (74.6)
Economic asset category*	
Low	43 (28.7)
Medium to High	107 (71.3)
Screened positive for depression (Hopkins Symptoms Checklist)	
No	111 (74.0)
Yes	39 (26.0)
Health status (missing = 7)	
No serious illness	134 (93.7)
Serious illness	9 (6.3)
Self reported alcohol consumption in past 30 days	
None	119 (79.3)
Some	31 (20.7)
PEth	
Negative	94 (62.7)
Positive (≥8 ng/ml)	56 (37.3)

*Low = bottom 2 quintiles.

### Alcohol consumption

Overall, 21% reported consuming alcohol in the past 30 days, but 37% tested positive for PEth. Of those reporting no alcohol consumption in the prior 30 days, 25% were positive for PEth (Table 2). Thirty-one patients reported use of alcohol and of these, 4 or 13% of them tested negative for PEth. Among the 60 drinkers (by self-report and/or PEth results), 31 reported having consumed alcohol, giving self-report a sensitivity of 48.2% (95% CI 34.7, 62%). The sensitivity by sex was 48.4% (95% CI 30.2, 66.9) for the men and 48% (95% CI 27.8, 68.7) for the women and the two were not statistically different from each other (Fisher's exact 2-sided p-value = 1.0).

**Table 2:** Sensitivity for self reported alcohol use, overall and by sex.

	*PEth* results		
Self report alcohol use, prior 30 days	Positive	Negative	Total
**Overall, n = 150**			
**Yes**	27 (48.2)	4 (4.4)	**31**
**No**	29 (51.8)	90 (95.6)	**119**
**Total**	**56**	**94**	**150**
**Among women, n = 98**			
**Yes**	12 (48.0)	4 (5.5)	**16**
**No**	13 (52.0)	69 (94.5)	**82**
**Total**	**25**	**73**	**98**
**Among men, n = 52**			
**Yes**	15 (48.3)	0 (0.0)	**15**
**No**	16 (51.7)	21 (100)	**37**
**Total**	**31**	**21**	**52**

### PEth levels by self report

Among those testing PEth positive, the median PEth value was higher among those reporting alcohol use (median = 477) compared to those who did not report any use of alcohol (median = 135.5) in the prior 30 days (Wilcoxon rank sum test p = 0.02, Table 3). The difference in the medians was statistically significant among the men (p = 0.01) but not the women (p = 0.14).

**Table 3:** PEth results among those with PEth>8 ng/mL, by self-reported alcohol consumption and by sex.

Self report alcohol use, prior 30 days	Median PEth ng/mL (25^th^, 75^th^ percentile)	Wilcoxon rank sum test (Self report yes vs no)
**Overall**		
Yes, n = 27	477.0 (51.8, 850)	
No, n = 29	135.5 (31.5, 322)	*z* = −2.43, *p* = 0.02
Total, n = 56	**191.3 (47.4, 683)**	
**Among women**		
Yes, n = 12	111.2 (34.5, 608.8)	
No, n = 13	31.5 (15.9, 116.0)	*z* = −1.46, *p* = 0.14
Total, n = 25	**51.8 (19.2, 432.5)**	
**Among men**		
Yes, n = 15	733.5 (338.5, 1228)	
No, n = 16	191.3 (132.5, 360.3)	*z* = −2.53, *p* = 0.01
Total, n = 31	**351.5 (136.0, 850.0)**	

### Predictors of under-reporting

Age, level of education, health status and history of depression were not associated with alcohol under-reporting in any of the bivariate logistic regression analyses (Table 4). In the entire sample, the odds of under reporting are almost three fold among men compared to women (OR = 2.93, 95%CI 1.26, 6.65), and even higher when the analysis was restricted to those not reporting any drinking (OR = 4.04, 95% CI 1.67, 9.74). The odds of under-report were 60% lower in the high/medium asset category compared to the low one (OR = 0.41, 95% CI 0.17, 0.94). Among drinkers, there was no significant association between gender and under-report (OR = 1.33 and 95% CI 0.48, 3.62). The odds of under-reporting were lower among those with partial literacy compared to no ability to read a sentence in the entire sample (OR = 0.17 and 95% CI 0.03, 0.87) and among those not reporting drinking (OR = 0.17 and 95% CI 0.03, 0.89).

**Table 4:** Bivariate logistic regression to determine the predictors of under-reporting alcohol use.

Characteristic	Under-report* in the entire sample (29 of 150) OR(95% CI)	Under-report* among drinkers (Self-reported and/or PEth positive) (29 of 60) OR(95% CI)	Under-report* among those not reporting drinking (29 of 119) OR (95% CI)
Gender			
Female	1.00	1.00	1.00
Male	**2.93 (1.26, 6.65)**	1.33 (0.48, 3.62)	**4.04 (1.67, 9.74)**
Age			
18–30	1.00	1.00	1.00
31–45	1.12 (0.43, 2.64)	0.94 (0.31, 2.85)	1.11 (0.44, 2.84)
Over 45	1.74 (0.56, 5.55)	2.36 (0.48, 11.72)	1.64 (0.51, 5.31)
Education			
None	1.00	1.00	1.00
Primary school	0.42 (0.16,1.14)	0.45 (0.12, 1.68)	0.41 (0.15, 1.16)
Secondary and above	0.39 (0.13, 1.23)	0.38 (0.09, 1.67)	0.40 (0.12, 1.31)
Literacy			
Cannot read a sentence	1.00	1.00	1.00
Read parts of a sentence	**0.17 (0.03, 0.87)**	0.18 (0.03, 1.19)	**0.17 (0.03, 0.89)**
Read a whole sentence	0.41 (0.16, 1.01)	0.42 (0.13, 1.43)	0.40 (0.15, 1.04)
Own land			
No	1.00	1.00	1.00
Yes	0.62 (0.27, 1.38)	0.67 (0.24, 1.86)	0.59 (0.255, 1.38)
Electricity in the home			
No	1.00	1.00	1.00
Yes	1.26 (0.46, 2.82)	1.09 (0.35, 3.44)	1.18 (0.45, 3.03)
Economic asset category			
Low	1.00	1.00	1.00
Medium to High	**0.41 (0.17, 0.94)**	0.58 (0.21, 1.67)	**0.35 (0.14, 0.85)**
Screened positive for depression (Hopkins Symptoms Checklist)			
No	1.00	1.00	1.00
Yes	0.88(0.34, 2.27)	0.91 (0.28, 2.95)	0.88 (0.33, 2.31)
Health status (serious illness)			
None	1.00	1.00	1.00
Present	1.13 (0.22, 5.76)	1.78 (0.15, 20.86)	0.97 (0.18, 5.12)
*Under-report defined as PEth+ but no self-reported drinking in the prior 30 days			

### Quantitative results

The median values for PEth and for self-reported measures of alcohol consumption among those reporting any alcohol consumption are reported in Table 5, along with the Spearman correlations between self-reported alcohol consumption and PEth. PEth was significantly correlated with the total number of days of drinking in the past 30 days (Spearman correlation  = 0.73, p<0.01), the total number of drinks in the past 30 days (Spearman correlation  = 0.72, p<0.01), and the amount of money spent on alcohol (Spearman correlation  = 0.43, p = 0.02).

**Table 5:** Measures of alcohol consumption and correlations with PEth, among those reporting any alcohol consumption, prior 30 days (n = 31).

Measure of alcohol consumption	Median (25^th^, 75^th^ percentile)	Spearman correlation with PEth value	p value
PEth (ng/mL)	405.5 (37.1, 796.5)	–	–
Number of days drinking	3.0 (2.0, 8.0)	0.73	<0.01
Typical number of drinks per drinking day*	3.5 (2.5, 7.0)	0.35	0.09
Total number of drinks (# days drinking x # drinks per drinking day)*	10.5 (6, 40)	0.72	<0.01
Total amount of money spent on alcohol (USD)	4.0 (1.2, 10)	0.43	0.02
Number of days drinking until uninhibited or more intoxicated	0 (0,0)	0.24	0.19

*n = 24 due to missing values for those drinking from shared or non-standard vessels.

## Discussion

Our study used a sensitive and specific alcohol biomarker to demonstrate that a high proportion of clients initiating ART at an HIV clinic at a large urban center in Uganda under-report their alcohol consumption. At the time of study recruitment, HIV clinic patients received counseling for at least 2 weekly sessions before initiation of therapy; this counseling included instructions to avoid alcohol consumption while on treatment. It is therefore not surprising that a significant proportion of patients under-reported their alcohol consumption. This is likely because of social desirability bias. Other authors have noted this concern. [14], [24] Our findings are consistent with our previous study in which we used %carbohydrate specific transferrin (%CDT), a biomarker for heavy alcohol use to examine under-reporting. [25] In that study, we found that 7% of those denying alcohol use were %CDT positive, a percent lower than found here but likely due to the low sensitivity of %CDT. [26] This study adds to the literature by using PEth, a more sensitive marker, demonstrating that 25% of those denying recent use were PEth positive and slightly over 50% of those who were PEth positive denied recent use.

Gender and economic asset index were significant predictors of under-reporting alcohol use overall and among those not reporting alcohol use. To the best of our knowledge, no other studies have demonstrated this before. Among drinkers, men were no more likely than women to under report. The high prevalence of alcohol use warrants screening for all patients, however the results suggest that men and particularly those with fewer assets may need more scrutiny for possible under-reporting if they deny any alcohol use. These patients may need reassurance that disclosure of alcohol use will in no way jeopardize their chances of initiating ART. Currently, clinics in Uganda do not have any strategies in place to reduce under-reporting. No other characteristic was associated with under-reporting alcohol use.

Our data also show a strong correlation between PEth and self-report measures of alcohol consumption among those who did report any alcohol consumption. The number of days of drinking in the past 30 days showed the strongest correlation (Spearman = 0.73) with the quantitative PEth result. This suggests that among those reporting any recent alcohol consumption, reporting of the frequency of consuming alcohol is highly valid, and also suggests that PEth may be a useful quantitative measure when self-report is unavailable or difficult to obtain. It is also notable that even among self-reported drinkers, the level of alcohol consumption was fairly low, with a median of 3 drinking days in the prior month. This is consistent with our previous findings of a large number of drinkers reporting reducing their alcohol consumption at the time of ART initiation in a previous wave of this same cohort [27]. Our data shows low correlation between number of days drinking until intoxicated and typical number of drinks per day with PEth probably because of under-report. However, the low correlation does not necessarily mean these measures are less useful.

PEth was detectable though results were significantly lower among those not reporting any alcohol consumption. This may imply that under-reporters may be lighter drinkers than those reporting drinking. In an analysis stratified by gender, the difference remained statistically significant among men but not the women. This may imply that women drinkers have similar levels of alcohol consumption whether they under-report or correctly report their alcohol consumption. However, this may be because of the small sample size. Also, because the women's PEth values are lower overall, even among those reporting alcohol use, their data represents the end of the scale where the difference is small. Regardless, the level of PEth was high among all those with detectable PEth, with a median of 191 ng/ml, which is well above the recent cutoff of 80 ng/ml which indicated drinking> = 4 drinks daily in a recent study among a group of patients with liver disease [28].

Our study has some weaknesses. PEth may not be completely sensitive and specific, therefore we may have under or over-estimated under-report. Secondly, there were some who reported drinking but were PEth negative, although these were very few. Our self-report measure spans 30 days while the biomarker spans 21 days; hence self-report may have reflected alcohol use beyond the window detectable by PEth. Also, it is still unclear whether PEth when assessed using LC/MS/MS is measuring any alcohol use or heavy alcohol use. [29] We took a conservative approach and used positive PEth results to suggest any alcohol use; however, it is possible that some PEth negatives may have consumed alcohol although moderately. This is supported by a recent study where participants with a negative PEth and reporting alcohol use were mainly light drinkers. [28]

Our study also warrants the necessity for clinicians to screen HIV infected patients initiating ART for alcohol use, especially in countries such as Uganda where alcohol consumption is high among drinkers. The high cost and limited availability of biomarkers restricts their use to research and limits their application in clinical settings.

In conclusion, our study has shown that many of HIV infected patients receiving ART under-report their alcohol consumption. Clinicians should screen the men more for possible under-reporting of alcohol consumption. Interventions depend on the reporting of alcohol use, therefore future research should develop ways to increase self-report.
